# Draft Genome Sequence of Root-Colonizing Bacterium *Bacillus* sp. Strain PTS-394

**DOI:** 10.1128/genomeA.00038-14

**Published:** 2014-02-13

**Authors:** Junqing Qiao, Youzhou Liu, Xuejie Liang, Yonghong Hu, Yan Du

**Affiliations:** a Institute of Plant Protection, Jiangsu Academy of Agricultural Sciences, Nanjing, China; b Biotechnology and Pharmaceutical Engineering, Nanjing University of Technology, Nanjing, China

## Abstract

Here, we report the high-quality draft genome sequence of *Bacillus* sp. strain PTS-394, isolated from the rhizosphere of tomatoes grown on Putuo Mountain (Xiamen, Fujian province, China), which exhibited excellent colonization ability on plant roots. The 4.0-Mb genome uncovered the mechanism for its potential root colonization ability and may provide novel insights into plant-bacterium interactions.

## GENOME ANNOUNCEMENT

Plant growth-promoting rhizobacteria (PGPR) can colonize on the roots of plants and improve plant growth (1). Several *Bacillus* species as members of PGPR have been studied extensively, and they possess the ability to suppress microbial plant pathogens and enhance the yields of crop plants (2) by colonizing on plant roots (3), producing antimicrobial secondary metabolites (4) and plant growth-promoting compounds (5, 6). *Bacillus* sp. strain PTS-394 was isolated from the rhizosphere of tomatoes grown on Putuo Mountain in China and was identified as a strain of *Bacillus subtilis* by 16S rRNA and *gyrB* gene sequencing. The strain showed excellent colonization ability on tomato roots and serves as an important biocontrol agent against tomato soil-borne diseases (7). Here, we analyzed its genome sequence to explore its genomic features as a PGPR.

Genome sequencing was performed with the Illumina/Solexa HiSeq 2000 at Shanghai Majorbio Bio-pharm Technology Co. Ltd. (Shanghai, China). In total, 1.3 Gb of clean data (12,606,050 paired-end reads with a 100-bp length, 500-bp library, and 314.9× coverage) was obtained for assembly after filtering out the low-quality reads. The total paired reads were *de novo* assembled with SOAP*denovo* (version 2.0) (8). All generated reads were assembled into 40 contigs (N_50_, 471, 675 bp) and rearranged in order into 29 scaffolds. All the contigs are >500 bp and the largest contig is 400.3 kb. A total of 4,128 coding sequences were predicted by Glimmer and annotated using information from RAST, GenBank, Pfam, and KEGG. Of these, 2,982 protein-coding sequences were assigned to Clusters of Orthologous Groups (COG) families (9, 10).

The draft genome of strain PTS-394 consists of 4,006,352 bases, with a 43.7% G+C content and 33 tRNAs. The genome includes several genes related to the biosynthesis of antimicrobial products that are similar to those in the genome of *B. subtilis* 168 (11). There are five gene clusters related to lipopeptide and polyketide synthase in the PTS-394 chromosome, such as surfactin, plipastatin, bacillibactin, bacilysin, and bacillaene. Additionally, extracellular phytase- and 2,3-butanediol synthesis-related genes are presented in PTS-394, which are responsible for plant growth promotion (6, 12). PTS-394 has putative biofilm formation-related genes that encode exopolysaccharides and γ-polyglutamate synthetase, which is a major element in the root colonization of PGPR (13, 14). In conclusion, the genome analysis of strain PTS-394 further demonstrates that it possesses the potential to be used as a biocontrol agent and helps us to study PGPR-plant interactions in the root ecological niche.

### Nucleotide sequence accession number.

The draft genome sequence of *B. subtilis* PTS-394 has been deposited at DDBJ/EMBL/GenBank under the accession no. AWXG00000000. The version described in this paper is the first version.
